# Temporal trends in birthweight and its association with overweight/obesity in schoolchildren from a Brazilian city: a cross-sectional panel study (2002–2019)

**DOI:** 10.1590/1516-3180.2025.3474.24042026

**Published:** 2026-09-21

**Authors:** Camila Elizandra Rossi, Bernardo Paz Barboza, Patrícia de Fragas Hinnig, Francisco de Assis Guedes de Vasconcelos

**Affiliations:** I Professor of the Undergraduate Course of Nutrition, Universidade Federal da Fronteira Sul (UFFS), Realeza (PR), Brazil. Permanent professor of the Postgraduate Program in Sciences Applied to Health, Universidade Estadual do Oeste do Paraná (Unioste), Francisco Beltrão (PR), Brazil.; II Student. Center for Public Health Research, Università degli Studi di Milano-Bicocca, Monza, Italy.; III Permanent Professor of the Postgraduate Program in Nutrition, Universidade Federal de Santa Catarina (UFSC), Florianópolis (SC), Brazil. Professor of the Department of Nutrition, School of Health Sciences, Universidade de Brasília (UnB), Brasília (DF), Brazil.; IV Permanent Professor of the Postgraduate Program in Nutrition, Universidade Federal de Santa Catarina (UFSC), Florianópolis (SC), Brazil.

**Keywords:** Infant, Low Birth Weight, High Birth Weight Infant, Child, Nutritional Status, Cross-Sectional Surveys, Birth weight, Overweight, Obesity, Associated Factors, Cross-Sectional Panels, Temporal Trend

## Abstract

**BACKGROUND:**

Long-term metabolic complications in childhood are associated with birthweight.

**OBJECTIVES:**

This study aimed to examine trends in the prevalence of low and high birthweight over a 16-year period and to investigate the association between birthweight and overweight/obesity among schoolchildren aged 7 to 10 years.

**DESIGN AND SETTING:**

The analyses included four school-based cross-sectional surveys conducted in Florianópolis.

**METHODS:**

The probabilistic panels were conducted in 2002, 2007, 2012–2013, and 2018–2019. Multiple logistic regression models were used to assess the associations between low and high birthweight and childhood overweight/obesity. Adjusted models included biological, socioeconomic, survey-period, and dietary-intake variables.

**RESULTS:**

Over the 16-year period, low birthweight showed a progressive and statistically significant increase, rising by 58.3% from 2002 to 2018–2019. In contrast, the prevalence of high birthweight was 9.1% in 2002 and declined significantly over the ten-year period from 2002 to 2012–2013, decreasing by 40.7%. In the adjusted model, high birthweight was significantly associated with overweight/obesity across all study waves (OR = 1.63; 95% CI = 1.32–2).

**CONCLUSION:**

A statistically significant upward trend in the prevalence of low birthweight was observed among schoolchildren in Florianópolis from 2002 to 2018–2019. Despite a reduction in high birthweight over time, high birthweight remained directly associated with overweight/obesity, independently of food intake and other confounding factors.

## INTRODUCTION

Birthweight is an important indicator of maternal and early childhood health. Regardless of gestational age, newborns weighing less than 2,500 g are classified as having low birthweight (LBW),^1^ whereas those weighing 2,500 g to 2,999 g are considered to have insufficient birthweight.^2^ Causes of LBW include prematurity, maternal undernutrition, and a range of intrauterine complications.^1-3^ Data from 158 countries and regions indicated that the prevalence of low birthweight in Latin America and the Caribbean was 9.6% in 2020.^4^ Estimated trends for Latin America and the Caribbean between 2000 and 2015 indicated only a marginal decline, from 8.8% to 8.7%.^5^ In Brazil's capital and its 26 states, the prevalence of low birthweight remained stable between 1996 and 2011, averaging approximately 8% annually.^6^

High birthweight (HBW), defined as birthweight greater than 3,999 g,^7^ is also of concern, as it reflects the presence of maternal metabolic risk factors such as gestational diabetes mellitus,^8^ pregestational obesity,^9^ or obesity during pregnancy.^10^ The reported prevalence of HBW in Northern Hemisphere countries ranges from 5.1% in Spain to 19.2% in Ireland,^11^ highlighting substantial geographical variation. In Brazil, estimates of high birthweight have also varied across municipalities, with rates of 11.9% in Recife^12^ and 7.3% in Florianópolis.^13^ This heterogeneity underscores the influence of regional demographic, nutritional, and healthcare factors on HBW prevalence. Trends in high birthweight have been only scarcely documented in the scientific literature. A study conducted in Southern India reported that the prevalence of newborns classified as large for gestational age (LGA) remained stable, with rates of 8.6% in 1996 and 8.8% in 2010.^14^

Long-term metabolic complications associated with low birthweight may manifest as early as childhood and include hypertriglyceridemia, dyslipidemia, fasting hyperglycemia, and an increased risk of type 2 diabetes mellitus,^1,15,16^ conditions commonly linked to obesity and excessive adiposity. Conversely, obesity and excessive adiposity are adverse long-term outcomes directly associated with high birthweight,^11,17 -18^ in addition to heightened risks of breast cancer, psychiatric disorders, hypertension, and both type 1 and type 2 diabetes.^19^ The literature search identified no studies that had examined temporal trends in the prevalence of low or high birthweight and their association with overweight or obesity later in life. Existing analyses have primarily focused either on trends in birthweight alone or on associations between birthweight and overweight/obesity or other metabolic complications, typically using cross-sectional designs that do not account for temporal variation.

## OBJECTIVE

This study aimed to examine temporal trends in the prevalence of low and high birthweight and their association with overweight/obesity from 2002 to 2019 among schoolchildren aged 7 to 10 years in Florianópolis. The study hypothesized that: (i) the prevalence of low and high birthweight remained stable over the 16-year period; and (ii) high birthweight was positively associated with overweight/obesity across the study period.

## METHODS

### Study design and population

Data were collected from schools located in Florianópolis, Southern Brazil. In 2024, the city had an estimated population of 576,361 inhabitants^20^ and a very high Municipal Human Development Index (HDIm = 0.847).^21^ Data were obtained from the Study on the Prevalence of Obesity in Children and Adolescents (Estudo da Prevalência da Obesidade em Crianças e Adolescentes, EPOCA), which comprises four school-based cross-sectional surveys conducted in 2002, 2007, 2012–2013, and 2018–2019. EPOCA is a probabilistic study designed to investigate temporal trends in obesity and associated factors among children and adolescents aged 7 to 14 years.^22-24^ For the present analysis, only data from students aged 7 to 10 years were included.

The sampling parameters were consistent across the four survey waves. In the first stage of sampling, schools were randomly selected from clusters stratified by geographic area (Centre, Continent, North, East, and South) and by school type (public or private). The second stage varied by survey year. In 2002, all classes within each selected school were included, and all children enrolled in the 2nd to 5th grades were invited to participate.^23,24^ In 2007, the sample was drawn proportionally to the distribution of students registered in the 2004 School Census of Florianópolis (53,595 individuals), stratified by residential area, school type, sex, and age group. In the 2012–2013 and 2018–2019 surveys, sample sizes were calculated using data from the 2010 and 2017 School Censuses, respectively, and based on expected overweight/obesity prevalences of 38% in 2012–2013 and 39% in 2018–2019. Sampling details for each wave are summarized in Table 1, with further methodological information available in previous publications.^25,26^

**Table 1:** Sampling details of the EPOCA surveys in 2002, 2007, 2012–2013, and 2018–2019, Florianópolis

Parameters	2002	2007	2012–2013	2018–2019
Population of 7–10-year-old schoolchildren in the municipality	28.395	25.619	19.172	27.110
Total number of elementary schools in the municipality	122	87	85	82
Total number of schools included in the survey†	16	17	30	30
Prevalence of overweight/obesity (%)	10	10	38	39
Sampling error (%)	2	2	3.5 (two-tailed) with a 95% CI	3.5 (two-tailed) with a 95% CI
Design effect	2	1.3	1.8	1.8
Additional (losses/refusals)	–	10%	10%	10%
Expected final sample	–	1.210	1.440	1.430
Total number of invited students	3.313	1.350	2.234	3.098
Total number of students investigated	2.936	1.232	1.423	922
Participation rate	88.6%	91.2%	63.7%	29.8%

† School units were drawn based on geographic region, type of administration (public and private), and number of students.

### Data collection and definitions

The inclusion criteria were: (i) attendance at school on the day of data collection; (ii) provision of written informed consent by parents or guardians; and (iii) provision of written informed assent by the student at the time of data collection (applicable only to the 2018–2019 wave). The exclusion criteria were missing data on birthweight, weight, or height.

In all four waves, birthweight data were reported by parents or guardians. This data collection strategy has been widely used in scientific literature.^27,28^ Shenkin et al.^27^ analyzed studies in which birthweight data were available from both parental recall and birth records. The comparison of 19 studies showed a high correlation (0.9; 95% confidence interval [95% CI] = 0.83–0.93), particularly in samples from high-income countries. In addition, the absolute effect size for the difference between recalled and recorded birthweight was 1.4 g, which did not represent a statistically significant difference in the pooled data.^27^ Similarly, Moreno-Galarraga et al.^28^ compared recalled data provided by 241 parents in Spain, when their children were 4 to 6 years old, with birth data from medical records and found an intraclass correlation coefficient of 0.95 (95% CI = 0.94–0.96). Even in older samples, self-reported birthweight data have been shown to be reliable in Norwegian participants aged 29 and 63 years. The kappa values between self-reported data and the medical birth registry were 0.63 and 0.71 for the respective age groups, indicating substantial agreement.^29^ Birthweight was categorized according to internationally recognized standards as follows: low (< 2,500 g), insufficient (2,500–2,999 g), adequate (3,000–3,999 g),^2^ or high (≥ 4,000 g).^7^ This categorization was applied to enable the evaluation of temporal trends over the 16-year study period.

In all four cross-sectional panels, weight and height were measured by a trained team following the World Health Organization (WHO) protocol.^30^ Children's weight status was classified according to body mass index (BMI)-for-age z-scores into two categories: "not overweight or obese" (z-score ≤ +1) and "overweight or obese" (z-score > +1).^31^ Data on age, sex, and school type were obtained from school records across all survey waves.

Food consumption was evaluated using qualitative, illustrated questionnaires. Researchers administered these questionnaires in all four seasons of each year and on all school days. In the 2002 cross-sectional panel, children completed the Typical Day Food Questionnaire (TDFQ), a paper-based pictorial instrument printed in color on A4 sheets. The questionnaire included 16 food illustrations distributed across five meals in chronological order (breakfast, morning snack, lunch, afternoon snack, and dinner), and children were instructed to mark the items they typically consumed in a day. This instrument was improved based on validation studies carried out in this age group. First, the food questionnaire was validated against the 24-hour recall method and showed moderate agreement in a sample of schoolchildren from Florianópolis, as assessed by concordance analysis,^32^ suggesting that this instrument provides information similar to that obtained from a 24-hour recall. Second, four food items were added to each meal, and the guiding question on recall time was changed to the previous day. Third, an additional meal was added: the evening snack before bed. Therefore, in the 2007 and 2012–2013 waves, food consumption data were collected using this new version, renamed the Previous Day Questionnaire (PDQ), which included 21 food items and/or groups.^32^ This version showed acceptable reproducibility and validity, with average sensitivity and specificity values of 70.2% and 96.2%, respectively, for 12 food items in three combined meals.^33^ In the 2018–2019 wave, food consumption data were obtained using the Web-CAAFE (Consumo Alimentar e Atividade Física de Escolares, or Food Consumption and Physical Activities of Schoolchildren) questionnaire. The Web-CAAFE was developed from the earlier paper-based instruments and has been tested for reproducibility, usability, and validity.^34^ The food consumption section comprised the six previously defined meals, with an animated character (avatar) assisting children in identifying the meal being assessed. For each meal, children could select from 31 images representing specific food items or groups displayed on a computer screen. Table 2 provides a comparison of the food items investigated in each wave.

**Table 2:** Food and beverage items by survey year, Florianópolis, 2002 to 2018–2019

Food and beverage		Survey year			
Item or food group		2002	2007	2012–2013	2018–2019
Dairy products	Milk/cheese	■	■	■	
	Milk		■	■	■
	Cheese		■	■	■
	Coffee with milk		■	■	■
	Yogurt	■	■	■	■
Fruits and vegetables	Fruits	■	■	■	■
	Green leaves	■	■	■	■
	Legumes				■
	Vegetable soup		■	■	■
	Fresh juice	■	■	■	■
Fast food and processed meat	Hamburger/pizza	■	■	■	
	French fries	■	■	■	■
	Sausages				■
	Fried snacks/hamburger/pizza/hot dogs				■
*In natura* animal protein	Beef/chicken	■	■	■	■
	Fish	■			
	Fish/seafood		■	■	■
	Eggs	■			■
Major sources of carbohydrates	Cheese bread				■
	Pasta/bread/crackers	■	■	■	
	Pasta				■
	Bread				■
	Breakfast cereal				■
	Rice	■	■	■	■
	Beans	■	■	■	■
	Manioc flour				■
	Corn/potato/mashed potato				■
	Cake without icing				■
Sweets	Cake with icing				■
	Candies	■	■	■	■
	Stuffed biscuits				■
	Chocolate				■
	Ice cream				■
Sugary drinks	Sodas/soft drinks	■	■	■	■
	Chocolate milk	■	■	■	■
Highly processed food	Packaged chips-type snacks		■	■	■
	Instant pasta		■	■	■
	Water				■
**Total**		**16**	**21**	**21**	**34**

A child was considered to have consumed a given food group if they reported consuming at least one item from that group in the survey instruments. Variables were grouped according to markers of healthy and unhealthy food consumption outlined in the Brazilian Dietary Guidelines.^35^ This resulted in the following binary variables (yes/no): (i) healthy food consumption—fruit intake and intake of legumes or vegetables; and (ii) unhealthy food consumption—intake of sweets and soft drinks.

Birthweight was considered the main independent variable, while overweight/obesity was treated as the outcome variable. Because biological characteristics and individual behaviors may directly influence weight status, variables related to food consumption and sex were included as control variables. With respect to socioeconomic factors, social determinants are known to shape access to health services and health-related information. Previous evidence indicates that 80% of Brazilian children from wealthier families attend private schools and that monthly family income is directly associated with the likelihood of enrolment in a private school.^36^ Given this characteristic of Brazilian schoolchildren, and because the "family income" variable had limited responses in our study, "school type" was incorporated into the multivariate model both as a control variable and as a proxy for family income.

### Statistical analysis and ethical approval

Statistical analyses were conducted using STATA version 18.0. Variables describing the sample—sex, type of school, birthweight, and weight status (BMI-for-age and sex)—were summarized as absolute and relative frequencies using the "svy" command, which accounts for the sampling weight of each cross-sectional wave.

The four databases were merged into a single database to analyze temporal trends in birthweight categories, which were described using prevalence estimates with 95% CIs for each survey year. Differences across years were assessed through visual inspection of the overlap of 95% CIs, interpreted as a conservative, descriptive approach rather than a formal statistical test of trend. These methodological procedures are similar to those used in previous studies, including those by Spanholi et al.,^37^ Nascimento et al.,^38^ and Medeiros et al.^39^

To assess the association between exposure and outcome variables, birthweight categories collapsed. Low birthweight and insufficient birthweight were combined into a single category (LBW/IBW). Bivariate logistic regression analyses were then performed to examine associations between birthweight, control variables, and weight status within each wave and across all waves combined (using the variable "year of study" as a control variable). Subsequently, multivariable logistic regression was used to estimate the adjusted association between birthweight and overweight/obesity across the study period.

The four waves (2002, 2007, 2012–2013, and 2018–2019) were approved by the Human Research Ethics Committee of the Universidade Federal de Santa Catarina (UFSC) under protocol no. 037/2002, 028/2006, CAAE 02713312000000121, and 87539718.1.0000.0121, respectively.

## RESULTS

**Figure 1:**
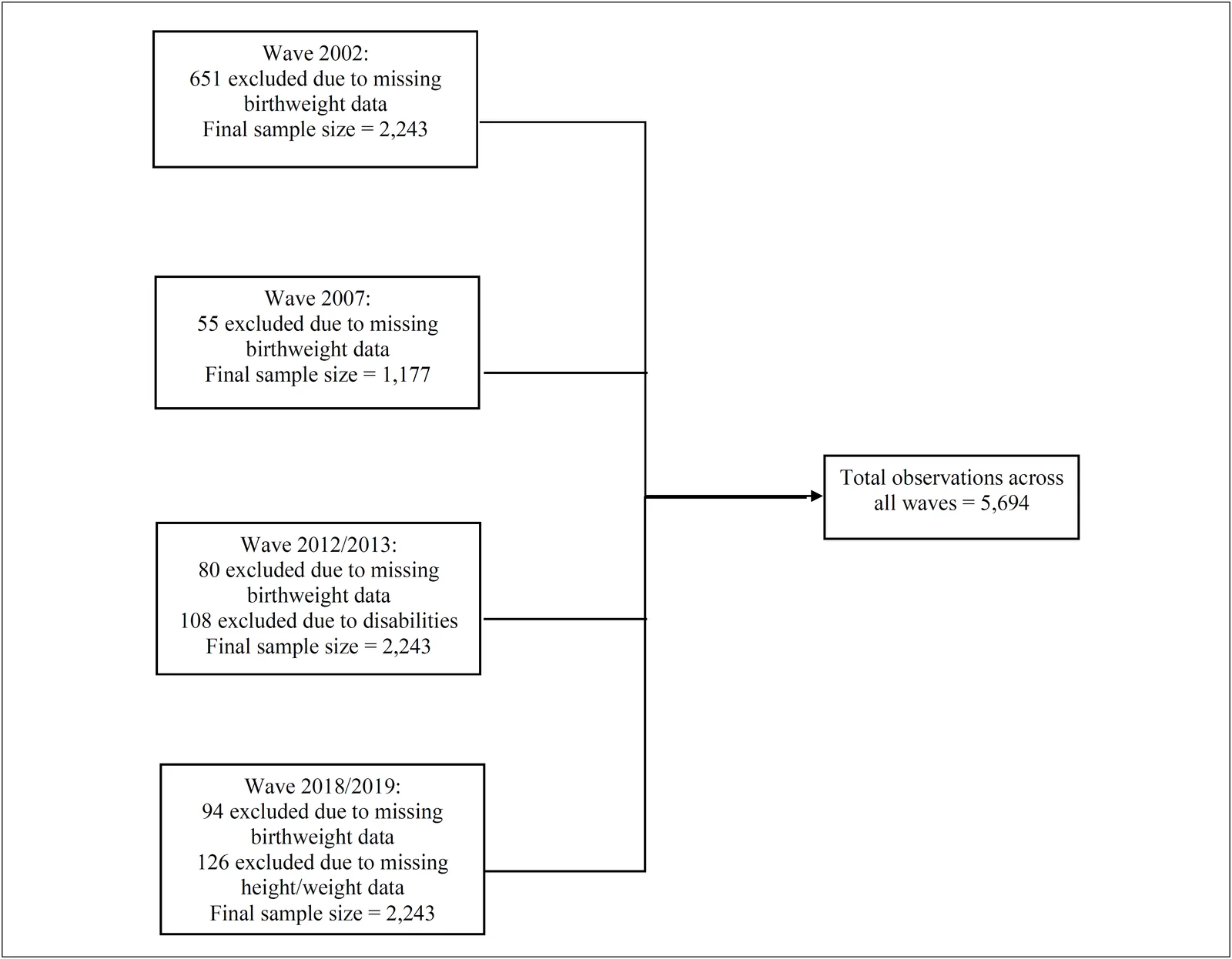
Excluded schoolchildren and the total sample in each study wave.

The final sample comprised 5,694 observations. Details on missing data and exclusions are shown in Figure 1.

The composition of each wave's sample was similar, with most participants being female and enrolled in public schools, and approximately one-third of the children having overweight/obesity. In 2007, the statistically significant reduction in the proportion of students from private schools required greater inclusion of students from public schools (Table 3).

**Table 3:** Sample characteristics in 2002, 2007, 2012–2013, and 2018–2019, Florianópolis

Characteristic		2002 (n = 2.243)			2007 (n = 1.177)			2012–2013 (n = 1.446)			2018/ 2019 (n = 828)			Final dataset (n = 5.694)		
		n	%/mean	95% CI^a^	n	%/mean	95% CI^a^	n	%/mean	95% CI^a^	n	%/mean	95% CI^a^	n	%/mean	95% CI^a^
Sex																
	*Male*	1.094	48.8	46.7–50.8	572	48.6	45.7–51.4	680	47	44.5–49.6	366	44.2	40.9–47.6	2712	47.6	46.3–48.9
	*Female*	1.149	51.2	49.2–53.3	605	51.4	48.5–54.3	766	53	50.4–55.5	462	55.8	52.4–59.2	2982	52.4	51.1–53.7
School type																
	*Public*	1.406	62.7	60.7–64.7	902	**76.6** *	**74.1–79**	930	64.3	61.8–66.7	498	60.1	56.8–63.4	3736	65.6	64.4–66.8
	*Private*	837	37.3	35.3–39.3	275	**23.4** *	**21–25.9**	516	35.7	33.3–38.2	330	39.9	36.6–43.2	1958	34.4	33.2–35.6
Birthweight (g)		2.243	3.313,3	3.229,2–3,397,5	1.177	3.300,4	3.211,7–3.389,2	1446	3.220,7	3.178,1–3.263,2	828	3.197,2	3.232,1–3.262,3	5694	3.264,8	3.250,2–3.279,3
Birthweight^c^																
	*LBW*	134	**6** *	**5.1–7**	97	8.2	6.8–10	141	**9.7** *	**8.3–11.4**	79	**9.5** *	**7.7–11.7**	451	7.9	7.2–8.6
	*IBW*	417	18.6	17–20.3	226	19.2	17.1–21.1	309	21.4	19.4–23.6	168	20.3	17.7–23.2	1120	19.7	18.6–20.7
	*ABW*	1.488	66.3	64.4–68.3	772	65.6	62.8–68.3	918	63.5	61–65.9	526	63.5	60.2–66.7	3704	65	63.8–66.3
	*HBW*	204	**9.1** *	**8–10.4**	82	7	5.6–8.6	78	**5.4** *	**4.3–6.7**	55	6.6	5.1–8.6	419	7.4	6.7–8
Weight status^d^																
	*Without OW/OB*	1.534	68.4	66.4–70.3	782	66.4	63.7–69.1	916	63.7	61.2–66.2	539	65.1	61.8–68.3	3707	66.3	65–67.5
	*With OW/OB*	709	31.6	29.7–33.6	395	33.6	30.9–36.3	521	36.3	33.8–38.8	289	34.9	31.7–38.2	1886	33.7	32.5–35

a 95% CI: 95% confidence interval.

c LBW: low birthweight; IBW: insufficient birthweight; ABW: adequate birthweight; HBW: high birthweight;

d OW: overweight; OB: obesity;

* bold values indicate prevalence estimates that are statistically significant according to confidence intervals.

The rate of low birthweight increased significantly, from 6% in 2002 to 9.7% in 2012–2013, representing a 61.7% increase. Although prevalence declined slightly in 2018–2019, it remained significantly higher than in 2002, with a 58.3% increase. In contrast, the prevalence of high birthweight declined significantly between 2002 and 2012–2013, with a 40.7% reduction. The prevalence of overweight/obesity rose from 2002 to 2012–2013, although this increase was not statistically significant (Table 3).

Bivariate analyses (data not shown) revealed a significant association between HBW and overweight/obesity, even when the variable "year of study" was included (OR = 2.21; 95% CI = 1.77–2.77). The strongest association was observed in the 2002 sample (OR = 2.4; 95% CI = 1.53–3.75). In addition, food consumption was significantly related to overweight/obesity, suggesting a direct influence on schoolchildren's weight status. For example, fruit consumption was inversely associated with overweight/obesity in two waves (2002 and 2018–2019) and when all waves were analyzed collectively (OR = 0.89; 95% CI = 0.80–0.98). Other variables, such as sex and school type, were also associated with the outcome. Therefore, all variables associated with overweight/obesity were included as control variables in the multivariate analysis.

Table 4 shows that, although the model was adjusted for food consumption variables, the association between HBW and overweight/obesity remained statistically significant across all waves (OR = 1.63; 95% CI = 1.32–2). Conversely, LBW showed an inverse association with the outcome, indicating that children born with low or insufficient birthweight had a reduced likelihood of having overweight/obesity (OR = 0.74; 95% CI = 0.65–0.85).

**Table 4:** Associations between birthweight and overweight/obesity from 2002 to 2018–2019, Florianópolis

Variables		Overweight/obesity^a^		
		OR	95% CI	p value
Birthweight				
	*Adequate birthweight*	Ref		
	*Low birthweight*	0.8	0.64–1	0.053
	*Insufficient birthweight*	**0.72**	**0.61–0.83**	**< 0.001**
	*High birthweight*	**1.62**	**1.32–2**	**< 0.001**
*Sex*				
	*Male*	Ref		
	*Female*	**0.73**	**0.65–0.82**	**< 0.001**
Type of school				
	*Public*	Ref		
	*Private*	**1.15**	**1.02–1.3**	**0.021**
Fruit intake				
	*No*	Ref		
	*Yes*	0.93	0.82–1.04	0.238
Legumes or vegetables intake				
	*No*	Ref		
	*Yes*	0.93	0.83–1.05	0.305
Soft drinks intake				
	*No*	Ref		
	*Yes*	0.96	0.85–1.09	0.573
Sweets intake				
	*No*	Ref		
	*Yes*	**0.8**	**0.71–0.91**	**< 0.001**
Survey year				
	*2002*	Ref		
	*2007*	1.08	0.93–1.27	0.285
	*2012–2013*	**1.22**	**1.05–1.42**	**0.008**
	*2018–2019*	1.05	0.87–1.28	0.548

a "With overweight/obesity" is the reference category;

OR: Odds ratio; 95% CI: 95% confidence interval; Ref: reference category. All variables had p < 0.20 in the simple models. Bold values denote statistical significance at the p < 0.05 level.

## DISCUSSION

The prevalence of low birthweight among schoolchildren from Florianópolis in 2002 was lower than the Brazilian rate (6% versus 8%). However, the temporal trend in the city showed a progressive and statistically significant increase over the following 10 years, with a 61.7% rise between 2002 and 2012–2013, exceeding national indicators. Although prevalence decreased slightly in 2018–2019, it remained statistically higher than in 2002, with a 58.3% increase. In contrast, the prevalence of high birthweight in Florianópolis was lower than that reported in other Brazilian cities and showed a statistically significant decline over the same period (2002 to 2012–2013), falling by 40.7%. Regarding overweight/obesity, approximately one-third of schoolchildren had this outcome over the 16-year period, with no statistically significant changes. The analysis of associations between birthweight and overweight/obesity revealed a statistically significant relationship between high birthweight and the outcome over the 16-year period, even after accounting for variables related to food consumption. Conversely, children born with low/insufficient birthweight were less likely to have overweight/obesity in childhood over the same period.

When compared with South American and Brazilian prevalence estimates in 2012, children in Florianópolis had a higher rate of low birthweight (8.6% and 8.3%, respectively, versus 9.7%). The higher prevalence and its increase in Florianópolis highlight the importance of determining whether greater efforts have been made to ensure the survival of small and preterm newborns. Pinheiro et al.^40^ analyzed survival rates in the first year of life among 90,153 newborns in Florianópolis and São José, finding a survival rate of 98.8%, which is high compared with national and international standards. Data from the National System of Live Births support this hypothesis: despite the increase in LBW prevalence in Florianópolis, mortality rates among children aged one year declined continuously between 2002 and 2018–2019 (from 9.8 deaths per 1,000 live births in 2002 to 6.6 per 1,000 in 2018–2019).^41^ Nonetheless, the underlying cause of rising low birthweight should not be overlooked and calls for strengthened monitoring of maternal health during pregnancy. Such care should be aligned with the principles of the Brazilian Policy for Integral Attention to Child Health Care within the Unified Health System, which includes a strategic focus on improving obstetric and neonatal care.^42^

The significant reduction in the prevalence of high birthweight in Florianópolis over ten years (from 9.1% to 5.4%) may indicate that maternal health during pregnancy has increasingly focused on glycemic control and maternal body weight. Data from 9,047,145 singleton births in South Korea between 2000 and 2020 also demonstrated a reduction, from 3.7% to 2.5%.^43^ Although the prevalence of HBW in Florianópolis is higher than that observed in South Korea, the rate recorded in 2018 remains considerably lower than national data from the United States of America (8.9%).^44^ In Brazil, pregnant women with diabetes are classified as being at risk within the public healthcare system and are eligible for more frequent and specialized treatment.^45^ In a study of 12,712 recent mothers in Brazilian hospitals, Rocha et al.^46^ found that women with diabetes received "more than adequate prenatal care" more frequently than women in other risk categories (74.1% versus 65%, respectively).

The association between HBW and overweight/obesity has also been reported in other Brazilian and international studies. In Santa Cruz do Sul, Rio Grande do Sul, birthweight greater than 3,000 g increased the likelihood of overweight/obesity between the ages of 7 and 14 years by 18%, even after adjustment for biological, socioeconomic, and behavioral factors.^47^ In another sample from Santa Cruz do Sul, Brand et al.^48^ evaluated 1,562 children and adolescents aged 6 to 17 years and identified the same positive association between birthweight and overweight (β = 0.84; 95% CI = 0.08–1.6) in models adjusted for both child and maternal characteristics. Among schoolchildren aged 9 to 11 years in São Caetano do Sul, São Paulo, Santos et al.^49^ found a positive and statistically significant association between birthweight and body mass index, as well as between birthweight and body fat (β = 0.001; 95% CI: 0.001–0.002, and β = 0.002; 95% CI: 0.001–0.003, respectively), in models that were also adjusted for physical activity levels. The findings on temporal trends indicate a statistically significant reduction in the prevalence of high birthweight over ten years, alongside stable rates of overweight/obesity among schoolchildren aged 7 to 10 years. Nevertheless, the association between high birthweight and overweight/obesity remained statistically significant in adjusted models across the 16 years. This suggests that the reduction in high birthweight prevalence over time was insufficient to lower the outcome at a population level. Public health efforts to prevent and manage the causes of birthweight ≥ 4,000 g are therefore essential. Future research to inform policy and practice in this field should consider trends in maternal health to identify the need for targeted interventions to manage glycemia and maternal weight status during pregnancy.

Despite the inverse association between low/insufficient birthweight and overweight/obesity, children born weighing more than 3,000 g remain at risk of developing other chronic conditions. In a meta-analysis of 28 studies, Martín-Calvo et al.^50^ reported that children born small for gestational age had a 2.33-fold higher risk of developing type 2 diabetes (95% CI = 1.05–5.17). The same meta-analysis also demonstrated that both LBW and being born small for gestational age were associated with increased insulin resistance.

This study has some limitations. First, the participation rate in the 2018–2019 wave was lower, mainly due to difficulties in obtaining parental consent and the length of the parental questionnaire; this could have introduced selection bias and may limit the representativeness of recent findings. Second, cross-sectional design does not allow for the assessment of cause-and-effect relationships. Third, the findings are context-specific and may not be generalizable to other settings, including Brazil as a whole or other countries. Notwithstanding these limitations, a comparison of non-response and response rates by school type in the third wave showed no differences between public and private schools,^37^ suggesting that the sample was homogeneous, at least with respect to socioeconomic factors. The study also has several strengths, including the representativeness of the data in the first waves, objective anthropometric measurements, and data collection by a trained team of anthropometrists. Since Florianópolis is a highly developed city, the results of this study may reflect the realities of other developed regions or countries. Regarding reported birthweight data, the city's developmental characteristics may be comparable to those observed in validation studies from high-income countries, which show high correlations between parental recall and birth records. Such maternal and neonatal health outcomes are likely connected to the implementation of, and population access to, high-quality healthcare services.

## CONCLUSION

This study identified a 61.7% increase in the prevalence of low birthweight over a 10-year period (2002 to 2012–2013) among schoolchildren in Florianópolis. Although a 3.4% decline was observed in the subsequent six years, the overall rise in low birthweight prevalence from 2002 to 2018–2019 remained statistically significant. In contrast, the prevalence of high birthweight showed a statistically significant reduction between 2002 and 2012–2013.

The association between birthweight and overweight/obesity remained statistically significant, even after accounting for food consumption variables and the temporal decline in HBW. These findings underscore the need for strengthened public health strategies to prevent macrosomia, given the association between HBW and the occurrence of overweight and obesity in childhood.

## Data Availability

The dataset generated for the analysis of the four waves of this study is not publicly available due to confidentiality and security restrictions, but is available from the corresponding author on reasonable request.
